# Development of the Big Ten Academic Alliance Collaborative for Women in Medicine and Biomedical Science: “We Built the Airplane While Flying It”

**DOI:** 10.2196/65561

**Published:** 2025-01-23

**Authors:** Maya S Iyer, Aubrey Moe, Susan Massick, Jessica Davis, Megan Ballinger, Kristy Townsend

**Affiliations:** 1 Department of Pediatrics, The Ohio State University College of Medicine, 420 Hamilton Hall, 1645 Neil Avenue, Columbus, OH, 43210, United States, 1 614-722-4384, 1 614-722-4380; 2 Nationwide Children's Hospital, Columbus, OH, United States; 3 Department of Psychiatry and Behavioral Health, The Ohio State University College of Medicine, Columbus, OH, United States; 4 Department of Dermatology, The Ohio State University College of Medicine, Columbus, OH, United States; 5 Office of Faculty Affairs, The Ohio State University College of Medicine, Columbus, OH, United States; 6 Department of Internal Medicine, The Ohio State University College of Medicine, Columbus, OH, United States; 7 Department of Neurosurgery, The Ohio State University College of Medicine, Columbus, OH, United States

**Keywords:** collaborative, gender equity, women in medicine, women in science, biomedical science, women, women+, gender, medicine, university, faculty, accessibility, career, equity, networking, opportunity, retaining, programming, Big Ten Academic Alliance, BTAA, academic alliance

## Abstract

Women-identifying and women+ gender faculty (hereto described as women+ faculty) face numerous barriers to career advancement in medicine and biomedical sciences. Despite accumulating evidence that career development programming for women+ is critical for professional advancement and well-being, accessibility of these programs is generally limited to small cohorts, only offered to specific disciplines, or otherwise entirely unavailable. Opportunities for additional, targeted career development activities are imperative in developing and retaining women+ faculty. Our goal was the development of a new collaborative of Big Ten Academic Alliance (BTAA) institutions to support gender equity for women+ faculty in medicine and biomedical sciences, with two initial aims: (1) hosting an inaugural conference and establishing a foundation for rotation of conference hosts across BTAA schools, and (2) creating an infrastructure to develop programming, share resources, conduct environmental scans, and promote networking. In 2022, leaders from The Ohio State University College of Medicine Women in Medicine and Science envisioned, developed, and implemented a collaborative named CommUNITYten: The Big Ten Academic Alliance for Women in Medicine and Biomedical Science. Conference program development occurred through an iterative and collaborative process across external and internal task forces alongside industry partners. We developed a fiscal model to guide registration fees, budget tracking, and solicitation of conference funding from academic and industry sponsors. Attendees completed postconference surveys assessing speaker or workshop effectiveness and suggestions for future events. Finally, we developed an environmental scan survey to assess gender equity needs and existing programming across BTAA institutions. In June 2024, The Ohio State University hosted the inaugural CommUNITYten conference in Columbus, Ohio, featuring 5 keynote presentations, 9 breakout sessions, and networking opportunities across one and a half days of curated programming. Nearly 180 people attended, with representation from 9 BTAA institutions, 6 industry companies, staff, and trainees. Postconference surveys showed 50% (n=27) of respondents were likely to attend another in-person conference and suggested future conference topics. The environmental scan survey launched in October 2024. We successfully established the CommUNITYten collaborative and hosted the inaugural conference. Establishing key stakeholders from each BTAA institution, obtaining sponsorship, and detailed conference planning and partnerships were critical in ensuring realization of this collaborative. The conference brought together leaders, faculty, staff, trainees, and industry partners from across the country and met the initial goal of networking, sharing resources, and building community for women+ faculty. These efforts lay a robust foundation for the BTAA CommUNITYten collaborative to foster ongoing collaboration, innovation, and progress in the years to come. Given the importance of steady improvements, this viewpoint may further guide the efforts of other individuals, groups, and leadership supporting women+ as they consider approaches and strategies advocating for gender equity at the national level.

## Introduction

Women-identifying and women+ gender faculty (women+ is inclusive of different identities pertaining to gender, hereto described as women+ faculty) in medicine and biomedical sciences face numerous barriers to career advancement. These barriers include fewer invitations to keynote speaking engagements, reduced academic promotion rates, decreased leadership opportunities, and disparities in research funding and other research metrics [1-4]. For women with intersectional identities, these barriers multiply [5]. There are few prominent medicine-based national career development programs in the United States targeted to women, such as the Association of American Medical Colleges (AAMC) Early-Career and Mid-Career programs, the Hedwig van Ameringen Executive Leadership in Academic Medicine (ELAM) program, the University of Michigan’s Rudi Ansbacher Advancing Women in Academic Medicine Leadership Scholars Program, and Harvard’s Career Advancement and Leadership Skills for Women, with metrics demonstrating postparticipation career success and advancement [6-8]. Women who participate in these career development programs are more likely than men and nonparticipant women to be promoted to associate professor, and as likely as men and more likely than nonparticipant women to be promoted to full professor within 10 years [9]. Career development program participation also improves retention of women in the academic workforce and leadership opportunities, while expanding participants’ networks and increasing visibility, both internally and externally at their institutions [10].

However, career development programs such as those from the AAMC and ELAM can be cost prohibitive and are often limited to one to two participants per institution [10]. Additionally, these programs do not accommodate faculty in medicine-adjacent fields such as biomedical engineering, veterinary medicine, and nursing. Attendance at shorter duration gender-specific conferences for women broadly in the medical and biomedical fields may provide similar benefits through delivery of content, networking, and professional support that are needed for career advancement [11]. Moreover, bridging the divide among health-related professions and bringing together women+ faculty across these fields may create a more effective cohort of professionals who can work synergistically to advance gender equity across academic institutions.

In 2022, leaders (all authors) from The Ohio State University College of Medicine’s (OSUCOM’s) Women in Medicine and Science (WIMS) created the Big Ten Academic Alliance (BTAA) collaborative for women in medicine and biomedical sciences with the goal of developing a network of women+ faculty in the BTAA to collaborate, share resources, provide support, and connect in a community that could be stronger together in combating gender barriers in these disciplines. Herein, we detail how we, as the leaders of this collaborative, developed a group of women+ individuals and respective institutions across the BTAA; how we crafted the inaugural conference; and how we are actively conducting an environmental scan survey and planning continuity of programming through conference host handoff, sharing of materials, and webinars.

## Collaborative Development

### Background and Overview

As a member of the Big Ten Conference, The Ohio State University (OSU) felt as if it was the natural place to start planning a collaborative to bring together peer institutions across the BTAA, an academic outcropping of this sports network. The BTAA was founded in 1958 [12]. Other BTAA conferences already exist in disciplines such as neuroscience; lipids; music education; women+ in technology; and Women in Science, Technology, Engineering, and Mathematics. The BTAA integrates across an existing network of deans, with central coordination and support in Rosemont, Illinois. The BTAA currently includes 14 universities. We chose to cast a broad net for the collaborative development and included institutions from all 19 prior, current, and future universities in the BTAA (Table 1). This BTAA collaborative of women in medicine and biomedical sciences would enable similar-sized universities to share effective programming and resources and work together to address academic gender inequity.

**Table 1.** The BTAA^a^ institutions.

	Member of BTAA	Representatives on ETF (n)^b^^,^^c^
Indiana University	✓	4
Michigan State University	✓	3
Northwestern University	✓	3
Pennsylvania State University	✓	3
Purdue University	✓	2
Rutgers University	✓	1
The Ohio State University	✓	4
University of California, Los Angeles	✓^d^	1
University of Maryland	✓	2
University of Nebraska-Lincoln	✓	3
University of Chicago	✓^c^	1
University of Illinois	✓	2
University of Iowa	✓	1
University of Michigan	✓	2
University of Minnesota	✓	2
University of Oregon	✓^d^	0
University of Southern California	✓^d^	1
University of Washington	✓^d^	1
University of Wisconsin-Madison	✓	1

a BTAA: Big Ten Academic Alliance.

b ETF: external task force.

c Historical member.

d New BTAA member.

As we generated the vision and mission for this initiative, we benefited by learning from prior speakers hosted by OSUCOM WIMS, as well as from internal advisors. Key suggestions from our advisors were to ensure early discussions with planning partners, include broad representation of organizing bodies, gather data that could be published as part of the initiative, and listen to key stakeholders. We differentiated ourselves from existing groups, such as the BTAA Women in Science, Technology, Engineering, and Mathematics and the AAMC Group on Women in Medicine and Science (GWIMS) by focusing on support of gender equity for faculty across medicine and biomedical sciences, with additional shared resources and interactions among trainees, staff, and relevant industry members.

We also envisioned that once the CommUNITYten collaborative was formed, this group could work together on initiatives that would benefit the BTAA’s future growth toward gender equity. Given the persistent national trend of women being promoted to full professor less frequently than men [1,13], we hope that this collaborative will provide future opportunities for networking, obtaining external talk and letters of evaluation for promotion, while also cultivating opportunities for networking, mentorship, collaboration, and sponsorship. Finally, we prioritized inclusivity and cultivating a partnership of interested industry companies, allies, staff, and leaders. Women faculty cannot solve the problems of gender inequity alone; we need the involvement and support of the entire community.

**Figure 1.**
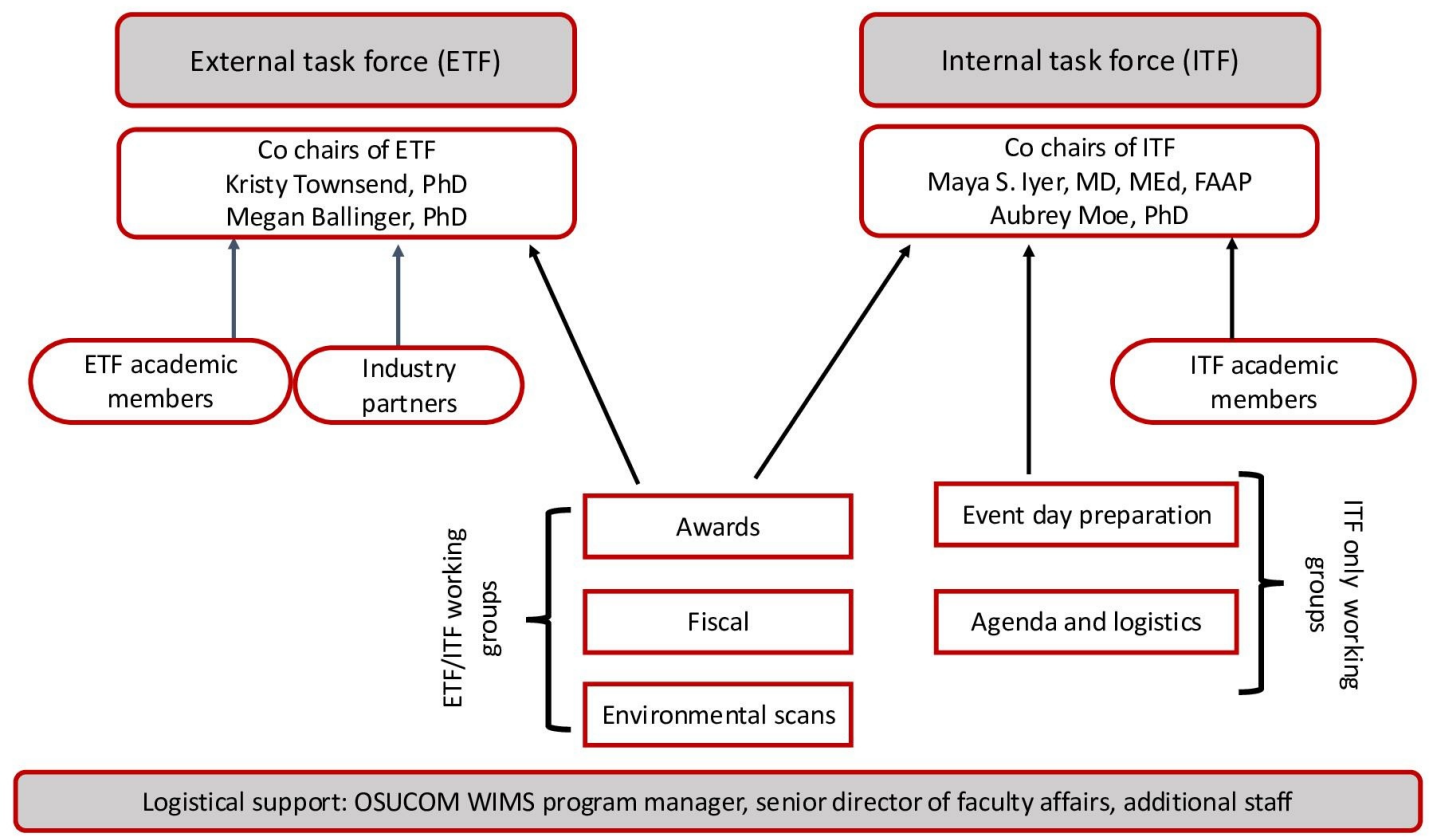
Reporting structure of BTAA CommUNITYten collaborative. BTAA: Big Ten Academic Alliance; ETF: external task force; ITF: internal task force.

To solicit membership to this collaborative’s leadership group, OSUCOM WIMS reached out to faculty representatives of the AAMC GWIMS to obtain contacts at the 19 BTAA institutions. Not all BTAA schools are part of GWIMS, so we also searched publicly available web pages dedicated to supporting women faculty at these institutions to find individuals who may be interested in planning the inaugural conference. OSUCOM WIMS also hosted a lunch roundtable at the 2022 AAMC Lead, Serve, Inspire annual meeting to gather additional participants [14]. In addition, we also relied on personal networks and connections to identify faculty members involved in initiatives that would align with the conference objectives. We were ultimately able to obtain at least one name and current contact information from 18 of the 19 BTAA institutions (Table 1). We included these individuals in all future email correspondence and meeting invitations. We also engaged with four industry representatives to bolster the networking component of this collaborative. These individuals together formed our external task force (ETF). We also created an internal task force (ITF) of faculty from OSU for development and planning for the inaugural conference. Figure 1 displays the CommUNITYten organizational chart.

### About ETF

The ETF began meeting by Zoom (Zoom Communications, Qumu Corporation) in June 2023. The ETF was led by two coauthors (MB and KT). During these calls, we refined the collaborative’s mission, solicited speaker votes for the initial conference, and discussed and voted on logistics such as registration fees, the cadence of ETF meetings, solicitation of sponsorships, and communication tactics.

### Industry Partners

In many cases, industry companies in pharma, biotech, or medicine are ahead of academics in their diversity, inclusion, and equity efforts. As we spoke with our four initial industry planning partners (Abbott, Bayer, Entrada, and Novartis), we learned that their efforts to educate on neurodiversity and to support women employee groups represented opportunities for academia to learn from industry. Furthermore, since academic-industry partnerships are key to successful academic medicine and biomedical research, and since these relationships can often be informal and dominated by men, we wanted to include industry attendees to bolster the networking goals of the conference. Industry could also benefit from learning about resources and best practices for gender equity in academic settings, and also benefit from networking opportunities for recruitment of potential employees. As part of our partnership with industry, we planned to have one panel discussion at the inaugural conference to learn more about industry equity initiatives, career opportunities, and translational research across academic-industry partnerships.

### About ITF

An ITF was specifically established to plan for the inaugural CommUNITYten conference and to execute key deliverables and tasks related to event logistics. This task force was led by two coauthors (MSI and AM). Given that OSUCOM WIMS led the establishment of the CommUNITYten collaborative, the ITF members were solicited from existing WIMS members, but invites were also extended to working groups from our academic sponsors and ETF members. The ITF met monthly and was further divided into working groups. This approach permitted work delegation to specific individuals and allowed for identification of specific leaders for each working group, providing leadership opportunities. A description of the five working groups is below. The ITF also worked closely with the WIMS’ program coordinator (coauthor JD), OSUCOM staff, and marketing teams.

## ITF Working Groups

### Agenda and Logistics

This working group focused on aspects of agenda planning, speaker communications, and coordination of timing for multiple tracks of speakers, seminars, and events during the day-and-a-half conference. Engagement and ongoing coordination with other working groups (fiscal and event day preparation) were critical to maintaining alignment of the agenda with CommUNITYten’s goals and budgetary resources.

### Event Day Preparation

This working group oversaw event logistics and worked directly under the WIMS program coordinator (coauthor JD). Specific items managed by this group included coordination of speaker transportation, audio or visual access and support, creation of program materials (including printed products and conference swag) and providing conference day assistance to attendees.

## ITF and ETF Joint Working Groups

### Awards

This group was led by two OSUCOM WIMS faculty members and had two additional BTAA members. Given the underrecognition of women recipients among societal and specialty-based awards [15-17], this working group intentionally developed three awards for the inaugural conference to recognize trainees and faculty at all academic ranks: (1) catalyst award, (2) rising star, and (3) luminary. The catalyst award is granted to a trainee (postdoctoral fellow, clinical fellow, or resident) who has demonstrated excellence, drive, and dedication in promoting women+ individuals in medicine and biomedical science or research. The rising star award recognizes early- or midcareer faculty (within 10 y of a full-time faculty position or rank of assistant professor) who have similar advanced women in their fields. The luminary award recognizes senior faculty members who have substantially contributed to the promotion and advancement of women+ individuals within or external to the nominee’s institution. Through an iterative process, this group developed these award descriptions, announcements, and scoring rubrics. These awards were presented at the inaugural conference.

### Fiscal

This working group developed the fiscal model to support the CommUNITYten conference and was coled by OSUCOM’s senior director of faculty affairs and coauthor KT in conjunction with the entire ETF. Starting with an initial US $20,000 investment from OSUCOM WIMS, the fiscal working group developed a registration model to generate needed revenue (with a fee schedule based on input and votes from the ETF), and solicited academic and industry sponsorships. Fundraising for the inaugural CommUNITYten conference involved a 3-pronged approach: (1) internal solicitation of host institution’s colleges and centers or institutes; (2) external solicitation of industry, nonprofit, or community organizations; and (3) academic sponsorship from other BTAA schools. Together, the fundraising from registrations and sponsorships would form the available budget for conference. Since we were fundraising as we were building the conference program, there was an iterative process whereby later fiduciary decisions (catering choices, for example) were dictated closer to the conference date and based on projections of confirmed incoming funds. By June 2024, we had raised approximately US $130,000 for the CommUNITYten collaborative.

To solicit sponsors, we generated a sponsor package that included an overview of the intent of the meeting and what would be gained by sponsoring across several options of sponsorship tiers (Multimedia Appendix 1). An Excel (Microsoft Corp) file was maintained to track sponsor calls, verbal commitments, and follow-up correspondence, or the “income” for the meeting. This file was compared to an Excel outline of expected expenditures (which would become a “budget,” once figures were finalized). The goal was to increase income above the numbers in the projected budget, in time for the deadline to finalize space and catering contracts. The last component of this effort was generating a dedicated account at OSU to hold funds separate from the college operating budget. This would allow for easy transfer of funds to the next host institution. We prioritized transparency in our efforts, with detailed recordkeeping and fiscal responsibility that was shared with the ETF. As an example of fiscal responsibility, our initial keynote choice had a prohibitively high speaker fee, and we now hope to have this speaker for a webinar in the future using remaining carryover funds now that the inaugural conference is over.

### Environmental Scan Surveys

This working group was led by two coauthors (MB and SM) in conjunction with the entire ETF. The group met monthly for 6 months to develop an institutional review board–approved research study protocol to ascertain current trends and faculty experiences across the BTAA institutions related to gender equity in medicine and biomedical science. The purpose for conducting a multi-institutional survey was to ask questions specifically on workplace climate, career promotion, leadership development, discrimination or bias, retention, mentorship, sponsorship, scholarship, pay equity, care responsibilities, and lactation or reproductive care services. This survey was piloted among non-BTAA faculty members to ensure understanding. The survey was launched in October 2024 and sent to conference participants or registrants, members of the ETF and other BTAA faculty contacts. Responses will be analyzed, and aggregated data will be shared among the participating BTAA institutions. Survey findings may identify opportunities for improvement, strategic interventions, and targeted programming that can benefit faculty across disciplines and shape programming initiatives for upcoming BTAA CommUNITYten conferences, future CommUNITYten collaborative work, and potentially a partnership with the BTAA and AAMC GWIMS going forward.

## CommUNITYten 2024 Inaugural Conference

The inaugural “CommUNITYten: Big Ten Academic Alliance Conference for Women in Medicine and Biomedical Science” was held on Friday June 7 and Saturday June 8 at the Ohio Union in Columbus, Ohio. Day 1 had 171 attendees and day 2 had 78 attendees, out of 220 total registrants. The final agenda for the conference is listed in Multimedia Appendix 2. The conference included 5 keynote presentations, 9 breakout sessions, coffee breaks with interactive activities (mindfulness, conversations surrounding people of difference, and a session with our Buckeye Paws service dogs), and a networking event on the first evening of the conference. Breakout sessions included presentations on topics relevant to the collaborative mission (eg, gender equity, developing a professional narrative, and effective science communication) as well as panel events on publishing, allyship from men, and using social media as a tool to establish professional presence. Finally, we awarded 1 catalyst, 1 luminary, and 2 rising star awards.

We conducted postconference evaluations (N=54 respondents). Among the respondents, on a 5-point Likert scale, 50% (n=27) reported that they would be likely to attend a future in-person BTAA CommUNITYten conference. In addition, 50% (n=27) stated that they would be likely to attend a webinar series offered by BTAA CommUNITYten. Nearly 60% (n=31) of respondents heard about the conference from a direct invitation. Open-ended comments showed that future topics should include negotiation; setting boundaries; navigating career changes; lesbian, gay, bisexual, transgender, or queer issues; creating networks; and clinical and translational research. Areas for improvement included shortening the conference to a one-day event, going beyond cis-normative topics, and including more time for networking.

## Challenges and Lessons Learned

The initial challenge in creating the CommUNITYten collaborative was “building the plane while flying it.” Our fiscal model was dependent on funds generated from registration costs, sponsorships, and the seed money from OSUCOM WIMS—all of which were unknowns when we initially embarked on this process. As a result, we had to establish our incoming funds flow simultaneously as we solicited speakers and panelists. We could not build a website until we had a set agenda. In addition, communicating with our ETF was challenging at the tail end of the academic year due to conflicts with scheduled meetings; loss of contacts (ie, individuals leaving their institutions due to retirement and promotions); and other academic obligations such as commencements, other conferences, and personal vacations.

We were fortunate to have the OSUCOM WIMS program coordinator (coauthor JD) serve as a project manager across all task forces and working groups. However, we recommend hosts of similar events bring on a full-time equivalent to support a host institution in planning a conference of this scale. Textbox 1 provides information on all the aspects of the conference that our program coordinator oversaw, including tasks carried out with our coleads, working group members, and support staff. Table 2 presents conference attendees’ institutional affiliations.

Textbox 1. **Conference planning and execution logistics. Logistical items listed were largely carried out by The Ohio State University College of Medicine (OSUCOM) Women in Medicine and Science’s program coordinator for the inaugural CommUNITYten conference, with some items carried out by coleads, OSUCOM support staff, or working group members.**

**Conference planning**

- Create an online file system (Microsoft Teams) to share information across internal and external partners.
- Schedule internal task force (ITF), external task force (ETF), marketing, and coleads meetings; send out reminder emails, meeting agendas, meeting notes, and postmeeting tasks.
- Research and secure space and catering orders.
- Preview facility or venue and work with venue event planner to confirm number of rooms, room layouts, technology needs, day of logistics, and build a timeline to meet venue deadlines.
- Collaborate with coleads and ETF and ITF on go/no go decisions such as event date or times, registration platform and wording, continuing medical education, fiscal model, Big Ten Academic Alliance (BTAA) logo usage, etc.
- Advertise through numerous channels, such as emails, campus-wide university newsletter, OSUCOM newsletters, and Twitter. Externally, the coleads advertised the conference through the Executive Leadership in Academic Medicine EDGE newsletter.
- Work with venue IT team for audio or video needs.
- Decide on and secure decorations (photo backdrop, centerpieces, or stage décor).
- Secure photographer.

**Fiscal**

- Schedule meetings for Women in Medicine and Science director and associate director to meet with The Ohio State University deans, center directors, and other leaders to request sponsorship.
- Meet with coleads and fiscal working group leads to review finances.
- Secure sponsor information: logo, marketing contact, fiscal contact, and contact for dissemination of information post conference.

**Speakers or agenda**

- Collate and track list of potential speakers, as determined by ETF, across price ranges and topics.
- Follow up on emails from coleads to potential speakers asking and securing availability, interest, and fees.
- Develop speaker intake forms (name, credentials, talk title and objectives, contact info, IT needs, travel needs, and payment forms).
- Track speaker information from intake through postevent payment process.
- Work with speakers on contracts and send to legal for approval.
- Set up logistics meetings with panelist groups and their moderators to understand their needs and desired speaker introductions.
- Set up logistics meetings with speakers and session presenters on talk time, topic, format, and logistics.
- Book speaker travel (lodging, flights, and transportation to the venue) and create individual speaker itineraries.
- Finalize agenda.
- Create day of speaker itineraries including travel, meetings, green room locations, and arrange shuttles or transport to and from the hotel to venue.
- Collect items for speaker gift bags to bring to the conference.
- Process speaker payments and honorariums, including receipts from speakers and create expense reports for travel reimbursement.

**Marketing**

- Create a comprehensive communication plan: Work with host institution marketing teams to create marketing language, create save the date PowerPoint (Microsoft Corp) slides, flyers, and email templates.
- Once approval is received from BTAA to use logo; work with BTAA representative to secure swag delivery before conference.
- Create registration site (Cvent) that includes deadlines, refund policy, option for single day registration versus full conference only, what information you want to gather from participants, what registration confirmation emails should look like, ability for registered participants to use an app or have a separate postregistration website, what sponsor logos will look like, what reports you will to need to monitor registration.
- Get input from coleads and event day logistics working group on aesthetic of Cvent site.
- Work with marketing team regularly to create and update QR codes, URL short links, content for social media posts, websites, and develop and pull daily reports from Cvent or conference registration website.
- Collaborate with marketing and event day preparation working group to install communications on electronic boards across institution.
- Add communications to electronic boards across institution.
- Create name tags.

**Working groups (awards, environmental scan, event day preparation, fiscal, and speaker or agenda and logistics**)

- Set up monthly group meetings, create or gather agendas, send reminder emails, and meeting notes and tasks.
- Develop a survey tool to capture award nominations and deadlines.
- Design and order plaques for the award recipients.
- Determine when and how to recognize awardees in event schedule with speaker or agenda working group.
- Market call for nomination emails to registered attendees and internally to participating colleagues.

**Day-of-conference logistics**

- Create run of show with day of assignments (décor, registration desk, speaker movement, monitor catering, audio-visual, space issues, and postevent clean-up).
- Inventory and gather supplies to take to venue (banners, signage, BTAA swag, speaker bags, BTAA notebooks, and pens).
- Create postevent surveys for feedback with coleads.
- Create rolling slide decks and master slide decks for each day, collaborate with audio-visual team at venue to ensure slide decks run smoothly.

***Postconference***

- Create and send thank you emails to speakers with coleads.
- Distribute postevent surveys for feedback for those that may have left early.
- Process venue or service-provider payments.
- Set up debrief meetings for ITF and ETF.
- Collate event survey data to send to coleads for analysis.

**Table 2.** Conference attendees’ institutional affiliations.

Group or institution	Friday June 7, 2024 (n)	Saturday June 8, 2024 (n)
Industry	6	0
Indiana University	3	4
Northwestern University	6	2
Pennsylvania State University	1	1
Rutgers University	2	2
The Ohio State University	134	56
University of Illinois	2	0
University of Maryland	3	3
University of Michigan	6	4
Univeristy of Nebraska	3	3
Non–Big Ten Academic Alliance institution	5	3
Total	171	78

After hosting the conference, we learned several lessons to improve future conference gatherings. First, future conferences in this series could benefit from selecting annual conference titles and themes to align content around specific goals. Many speakers expressed a desire for this information to tailor their talks to align with the conference’s intentions and goals beyond the broad gender equity focus. In addition, we learned the importance of engaging the institutional marketing team from the outset. Their support is crucial for successful outreach, within the institution and to external networks via LinkedIn. Before launching the registration platform, having a complete program agenda and speaker bios helps convert website visitors to registrants when they can see the full conference content. We also learned that shortening the conference to 1 day and avoiding programming on a weekend could boost attendance. Finally, if possible, we found that a 2-year timeline is needed to secure sponsorships and plan logistics associated with the in-person meeting.

## Future Directions

One of the pillars in the development of this conference was to establish a network of like-minded women+ interested in bolstering gender equity in medicine and biomedical science. In order to provide equitable leadership opportunities and accessibility of the in-person conference geographically, the ETF decided that the host institution would rotate every other year for in-person conferences. In the interim nonconference years, a virtual webinar series will continue the momentum and the networks established by this collaborative, with allocated budget voted on by the ETF and ITF from our rollover funds. We anticipate that each BTAA institution will be responsible for sponsoring a speaker for the interim year webinars. Additionally, we hope that results from the environmental scan, once completed, will be presented in upcoming meetings or publications and used as a needs assessment for future CommUNITYten conferences in determining conference themes, workshops, sessions, and speakers.

In summary**,** the BTAA CommUNITYten collaborative successfully launched in 2024. Establishing key stakeholders from each BTAA institution, obtaining sponsorship, and detailed conference planning and partnerships were critical in ensuring realization of this group. The inaugural conference brought together leaders, faculty, staff, trainees, and industry partners from across the country and met the initial goal of networking and developing interinstitutional community. Our future initiatives of conducting environmental scans and sharing resources are in progress, as well as development of a national webinar series. These efforts lay a robust foundation for the BTAA CommUNITYten collaborative to foster ongoing collaboration, innovation, and progress in the years to come.

## Supplementary material

10.2196/65561 Multimedia Appendix 1 Sponsorship tiers.

10.2196/65561 Multimedia Appendix 2 Conference agenda.

## Abbreviations

AAMC: Association of American Medical Colleges
BTAA: Big Ten Academic Alliance
ELAM: Executive Leadership in Academic Medicine
ETF: external task force
GWIMS: Group on Women in Medicine and Science
ITF: internal task force
OSU: The Ohio State University
OSUCOM: The Ohio State University College of Medicine
WIMS: Women in Medicine and Science
