# Supplementary material for: Development of the Big Ten Academic Alliance Collaborative for Women in Medicine and Biomedical Science: “We Built the Airplane While Flying It”
Source: JMIR Form Res. 2025 Jan 23;9:e65561. doi: 10.2196/65561 (PMC11781237; doi:10.2196/65561)
Supplement: Multimedia Appendix 1 [file formative-v9-e65561-s001.docx]

**BTAA Women in Medicine and Biomedical Research**

CommUNITYten Inaugural Conference – June 7-8th, 2024

The Ohio State University Union, Columbus, Ohio

**SPONSORSHIP TIERS**

| ***Sponsorship Level(s)*** | ***Fee(s)*** | ***Sponsor Benefits*** |
| --- | --- | --- |
| Academic Institution (or College) - Current or Future University in BTAA | 1. $5000 2. $10,000 3. $15,000 4. $20,000 | (A) 5 guaranteed tickets comp/gratis for your institution plus 5 held until Jan 15, 2024 to be purchased at early registration rate; Logo on program; Logo on meeting welcome slides;  (B) Same as A, with 10 tickets gratis and 5 held; ability to bring promotional materials; receives a copy of environmental scan survey results  (C) Same as A, with 15 tickets gratis and 5 held  (D) Same as A with 20 tickets gratis and 10 held |
| Partnering Industry Company | 1. $25,000 – Speaker Sponsor 2. $35,000 – Speaker Sponsor and Evening Event Sponsor 3. $55,000 – Speaker Sponsor, Evening Event Sponsor, and Tabling 4. Sponsorship lower than $25,000 is negotiable with the organizers, esp. for smaller companies | 1. 10 tickets comp/gratis plus 10 guaranteed tickets held at early registration rate for your company to purchase (held until 6wks prior to meeting), logo on program; logo on meeting welcome slides, Named speaker/workshop event as part of main program – to be advertised ahead of event 2. Same as A, plus named evening event on June 7 for conference attendees; 15 comp tickets plus 10 held 3. Same as B, plus tabling and distribution of promotional materials; 20 comp tickets plus 10 held 4. Negotiable – please contact wims@osumc.edu |

| ***Ticket Category*** | ***Registration Price*** | ***Details*** |
| --- | --- | --- |
| Academic Faculty | $350 | Attendance for both days, in person (includes breakfast and lunch on June 7^th^ and breakfast on June 8^th^), hotel discount code provided |
| Trainees (grad students, postdocs, medical students, residents, fellows) | $200 | Attendance for both days, in person (includes breakfast and lunch on June 7^th^ and breakfast on 8^th^); Limited scholarships available for reduced cost with industry sponsorship – see Cvent for details, hotel discount code provided |
| Industry Attendees | $600 | Attendance for both days, in person (includes breakfast and lunch on June 7^th^ and breakfast on 8^th^), hotel discount code provided |
