# Supplementary material for: Development of the Big Ten Academic Alliance Collaborative for Women in Medicine and Biomedical Science: “We Built the Airplane While Flying It”
Source: JMIR Form Res. 2025 Jan 23;9:e65561. doi: 10.2196/65561 (PMC11781237; doi:10.2196/65561)
Supplement: Multimedia Appendix 2 [file formative-v9-e65561-s002.pdf]

CommUNITYten Conference:
The Big Ten Academic Alliance Women in Medicine and Biomedical Science Conference

June 7 and 8 at The Ohio Union

June 7, 2024

8 a.m.	Registration and check-in<br>Archie M. Griffin Ballroom – East
8:45 a.m.	Welcome<br>Archie M. Griffin Ballroom – East
9 a.m.	First and foremost – the power of women leading in medicine & science<br>Archie M. Griffin Ballroom – East<br>*J. Nwando Olayiwola, MD, MPH*
10:10 a.m.	Creating connections: An introduction to the Alda Method (interactive workshop) – breakout session<br>Archie M. Griffin Ballroom – West<br>*Alan Alda*
10:10 -11:10 a.m.	Publishing panel breakout session<br>Archie M. Griffin Ballroom – East<br>*Mary Fristad, PhD*<br>*Bethany Moore, PhD*<br>*Loren Wold, PhD, FAHA*
11:20 a.m.	Equality is for everyone: How gender norms underpin our behavior at work and home – breakout session<br>Archie M. Griffin Ballroom – East<br>*Kate Mangino, PhD, Gender Expert*
12:30 p.m.	Lunch<br>Archie M. Griffin Ballroom – East
1:30 p.m.	Keynote: Ten lessons from a career studying physicians' careers<br>Archie M. Griffin Ballroom – East<br>*Reshma Jagsi, MD, PhD*
2:30 p.m.	Coffee break and networking<br>Archie M. Griffin Ballroom – East<br><br>A mindfulness break for the health care professional<br>Archie M. Griffin Ballroom – West<br>*Maryanna Klatt, PhD*<br><br>Mosaic conversations<br>Ohio State Inc, Founders Room<br>*Gloria Fleming, MD*<br><br>Buckeye Paws<br>Archie M. Griffin Ballroom – East

Inaugural host institution

BIG ACADEMIC ALLIANCE

THE OHIO STATE UNIVERSITY

COMD 24245450

3 p.m.	Allyship panel – breakout session<br>Student Alumni Council<br>*Nick Breitborde, PhD*<br>*Sara Childers, PhD*<br>*James MacDonald, MD, MPH*<br>*Leon McDougle, MD, MPH*<br>*John Pandolfino, MD*<br>*Timothy Pawlik, MD, PhD, MPH*<br>*Andreá Williams, PhD*
3 p.m.	Industry panel – breakout session<br>Archie M. Griffin Ballroom – West<br>*Julie Johnson, PharmD*<br>*Santha Ramakrishnan, PhD*<br>*Dominique Williams, MD, MPH*
3 p.m.	What if your world was a book? – breakout session<br>Archie M. Griffin Ballroom – East<br>*Sarah Lagrotteria*
4-6:30 p.m.	Networking event<br>Potter Plaza

June 8, 2024

7 a.m.	Registration and check-in<br>Archie M. Griffin Ballroom – East
7:45 a.m.	Welcome<br>Archie M. Griffin Ballroom – East
8 a.m.	Keynote: Moving the needle: strategies to support success, belonging and inclusion<br>Archie M. Griffin Ballroom – East<br>*Nancy Spector, MD*
9:10 a.m.	An introduction to researcher coaching (virtual lecture) – breakout Session<br>Archie M. Griffin Ballroom – West<br>*Katy Mahoney, PhD*
9:10 a.m.	Social media panel – breakout session<br>Archie M. Griffin Ballroom – East<br>*Nicole Baldwin, MD*<br>*Leslie Kim, MD, MPH*<br>*Nikki Sunstrum*<br>*Rachel Kearney, BSDH, PhD*
10:20 a.m.	How to build a successful academic department<br>Archie M. Griffin Ballroom – East<br>*Claire Verschraegen, MD*
11:30 a.m.	New-age effective teaching<br>Archie M. Griffin Ballroom – East<br>*Heather Hirsch, MD*
12:30 p.m.	Awards ceremony<br>Archie M. Griffin Ballroom – East

Scan for full event agenda and conference details.
